# Heat stress presenting with encephalopathy and MRI findings of diffuse cerebral injury and hemorrhage

**DOI:** 10.1186/1471-2377-13-63

**Published:** 2013-06-17

**Authors:** Waldo R Guerrero, Shaun Varghese, Sean Savitz, Tzu Ching Wu

**Affiliations:** 1 Department of Neurology, University of Texas Medical School-Houston, UT Health, Houston, TX, USA

**Keywords:** MRI, Heat, Stroke, Stress, Cerebellum, Ischemia

## Abstract

**Background:**

Heat stress results in multiorgan failure and CNS injury. There a few case reports in the literature on the neurological consequences of heat stress.

**Case presentation:**

We describe a patient with heat stress presenting with encephalopathy and bilateral cerebral, cerebellar, and thalamic lesions and intraventricular hemorrhage on MRI.

**Conclusion:**

Heat stress should be in the differential diagnosis of patients presenting with encephalopathy and elevated serum inflammatory markers especially if the history suggests a preceding episode of hyperthermia.

## Background

Heat stroke is a medical emergency resulting from a body temperature of greater than 40°C (104°F) usually resulting in alteration of consciousness [1,2] and associated with a 10%-50% mortality rate [3]. It usually results in multiorgan failure. However, the central nervous system including the cortex, cerebellum, basal ganglia, and anterior horn cells of the spinal cord are also vulnerable to hyperthermia [2,4].

There are a limited number of cases in the literature that describe the CNS injury related to heat stress. Previous reports of heat stress describe diffuse cerebellar atrophy [1], hyperintense lesions on diffusion-weighed imaging (DWI) in the dentate nuclei [5], bilateral superior cerebellar peduncles (SCPs), thalami [6], central tegementum of the midbrain [7], hippocampi, cerebellum, and cerebral cortices [8]. Herein, we report a patient with heat stress presenting with encephalopathy and bilateral cerebral, cerebellar, and thalamic lesions and intraventricular hemorrhage on MRI.

## Case presentation

A 66 year-old man with a past medical history of hypertension, hypothyroidism, hyperhomocysteinemia, and major depressive disorder was transferred to our hospital for further evaluation of altered mental status. The patient had been cattle ranching four days prior to his initial hospital presentation. While cattle ranching, he began having malaise and cramping in his hands. He subsequently vomited and started having diarrhea. Given that the temperatures outside were above 100 degrees Farhanheit during the first two days while cattle ranching, the patient’s wife felt he was dehydrated and encouraged him to drink more fluids. On the third day of cattle ranching, the patients’ mental status deteriorated as he became increasingly lethargic. He was taken to an outside hospital and was intubated for airway protection. Outside hospital laboratories demonstrated the following significant laboratories: WBC 11.4 K/cmm, Hemoglobin 15.6 K/cmm , Na 143 mEq/L, K 5.9 mEq/L, Phosphorus 6.4 mEq/L, anion gap 22 mEq/L, creatinine 5.5 mg/dL, C-reactive protein (CRP) 22.7 mg/L, erythrocyte sedimentation rates (ESR) 50 mm/hr, lactate 2 mMol/L, and troponin 0.229 ng/mL, CK-MB 8.1 ng/mL, and lipase 1091 unit/L. The patient displayed evidence of multiorgan failure and was transferred to our hospital for further care.

Upon arrival (five days after his initial presentation to the outside hospital), the patient was euthermic at 97.8 F. His heart rate was 86, blood pressure 162/82, and respiratory rate 18. His neurological examination was significant for lethargy, dysarthria, impaired vertical eye movements, diffuse weakness in all limbs (4 out of 5 power in the extensors of the arms and flexors of the legs), bilateral ataxia on finger to nose, and bilateral extensor plantar responses. General physical examination was normal other than bilateral scattered crackles on lung auscultation. There were no stigmata of infectious endocarditis.

Laboratory findings upon transfer were remarkable for an elevated C-reactive protein of >190 mg/L, ESR of 111 mm/hr, ALT 91 U/L, AST 51 U/L, and a macrocytic anemia with a hemoglobin and hematocrit of 13.2/38.2 K/cmm. WBC, platelets, PTT/PT/INR, vitamin B12, folate, homocysteine, cardiac enzymes including CK, thyroid studies, creatinine, and blood cultures were normal. Abdominal ultrasound showed evidence of hepatic steatosis and transthoracic echocardiogram showed normal ejection fraction with left ventricular hypertrophy and diastolic dysfunction but no evidence of vegetations or wall motion abnormalities.

**Figure 1:**
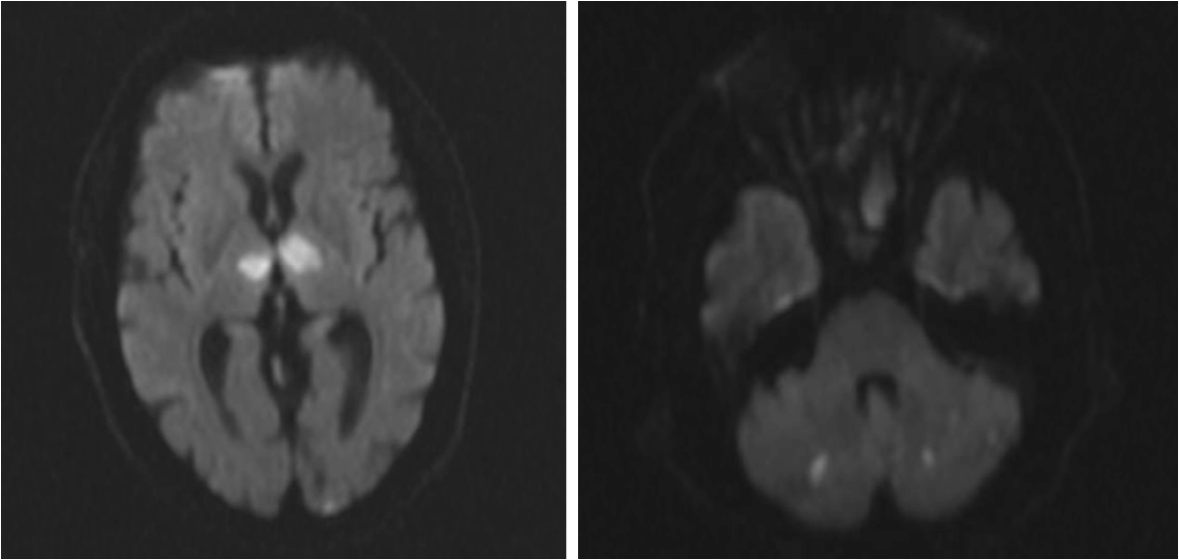
Diffusion weighted imaging showing multiple punctate foci of restricted diffusion involving the cerebellum bilaterally as well as two larger foci of the dorsomedial and ventrolateral thalami bilaterally.

**Figure 2:**
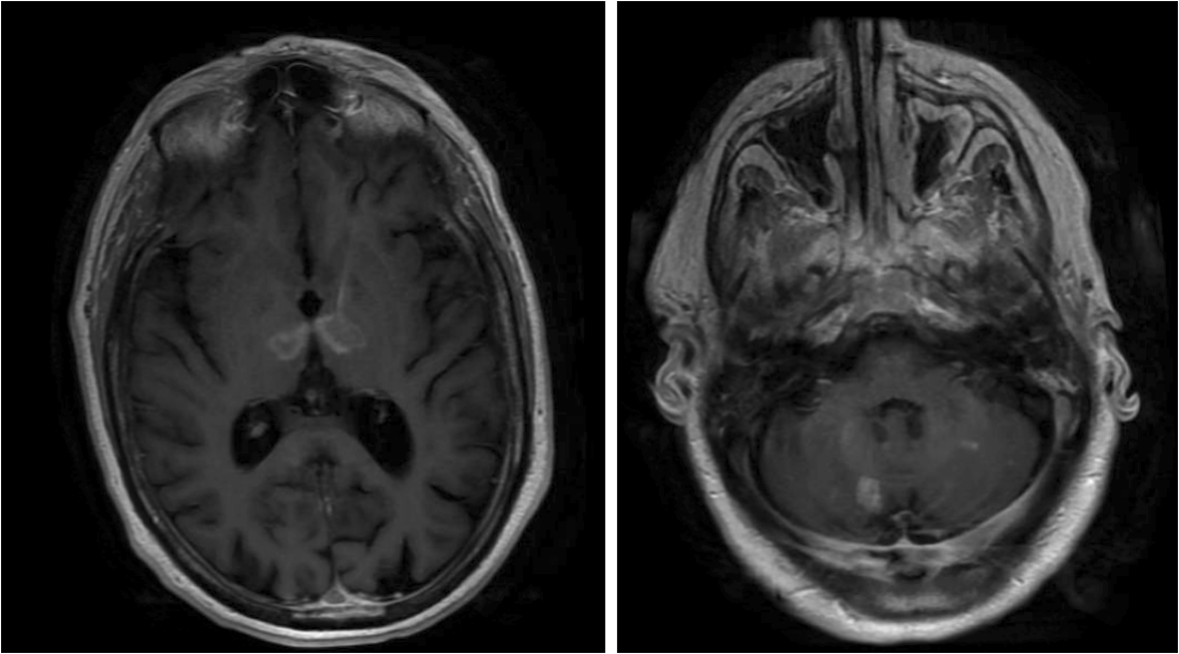
T1 post gadolinium study showing heterogeneous enhancement of cerebellar and thalamic lesions.

Diffusion weighted magnetic resonance imaging showed multiple punctate foci of restricted diffusion involving the frontoparietal cerebral cortex and cerebellum bilaterally as well as two larger foci in the dorsomedial and ventrolateral thalami bilaterally (Figure 1). These abnormal lesions showed decreased ADC and heterogeneous postcontrast enhancement (Figure 2). Magnetic resonance angiography of the head and neck vasculature showed normal flow-related signal within the intra and extracranial vessels. There was trace layering of intraventricular hemorrhage within the bilateral occipital horns on the GRE sequence.

After nine days of hospitalization, the patient started to awaken and became more alert. During hospitalization he was started on Amantadine and was eventually discharged to an acute rehabilitation facility. After two weeks of inpatient therapy, the patient had persistent cognitive deficits including poor short term memory.

## Discussion

When the core body temperature rises above 40°C, thermoregulation fails and multiorgan failure ensues. With the worsening of global warming, heat stroke or stress as a cause of morbidity will increase [9,10]. The pathophysiology behind heat stress is similar in nature to sepsis. During heat stress, blood is shunted from the splanchnic vasculature and is redirected to the periphery in order to dissipate heat. Cytokines are increased [9] and heat shock protein synthesis [9,11] is also increased in an acute-phase response. In addition, the body becomes increasingly deprived of volume and salt eventually leading to splanchnic ischemia and increased bowel permeability ultimately resulting in endotoxin release into the blood stream. This endotoxin release leads to a heightened inflammatory acute-phase response and sepsis-like picture [9].

Similar to our patient, previous heat stress case reports have shown that the most common initial presenting symptom is impaired consciousness [8,12,13]. In addition, patients have presented with multiorgan failure and systemic findings of intestinal dysfunction, rhabdomyolysis, elevated liver function tests and acute renal insufficiency [12,13]. This multiorgan failure is thought to result from the body’s effort to avoid a functional hypovolemia. A compensatory vasoconstriction of the

Splanchnic and renal vasculature occurs causing the symptoms of nausea, vomiting, and diarrhea. Although most of these systemic symptoms resolve within one week in these cases, the cognitive and memory deficits have persisted [12,13]. In addition, patients have had continued difficulty with fine motor coordination, ataxia/dysmetria, and difficulty with balance and coordination [12,13]. Our patient’s presentation is in line with these previous reports.

There are several proposed mechanisms by which heat stress leads to CNS injury. One mechanism involves the release of cytokines which increase the leakiness of the blood–brain barrier and blood-cerebrospinal fluid barrier thus resulting in vasogenic edema and neuronal death [9]. Pro-inflammatory cytokines can also directly cause apoptosis. Interestingly, our patient’s ESR and CRP were significantly elevated. These values may be surrogate markers supporting the mechanism of an exaggerated systemic inflammatory response in patients with heat stress.

Our patient also had evidence of CNS hemorrhage and postcontrast enhancement. A prior case report showed bilateral cerebellar lesions with probable blood products and gadolinium enhancement [12]. These findings were thought to be related to small-vessel ischemia. We argue that these changes actually may instead support the mechanism of cytokine-mediated leakiness of the blood–brain barrier. Another mechanism might involve augmented hemostasis and microvascular disease leading to small vessel ischemic change [9,10]. Pro-inflammatory cytokines could also activate endothelia to become adhesive, a phenomenon known as Shwartzman reaction. This is a rare reaction of the body to endotoxins leading to thrombosis in the affected tissue [14].

Finally, there is a direct toxic effect of heat on the CNS especially on cerebellar Purkinje cells [10,11]. In animal models, purkinje cells are thought to have the highest concentration of heat shock proteins [11]. Interestingly, loss of Purkinje cells residing in the cerebellar cortex due to hyperthermia have been reported in neuroleptic malignant syndrome [15]. Postmortem studies show cerebellar atrophy pointing toward the sensitivity of Purkinje cells to heat injury.

Regarding the lesions in the thalami and cortex in our patient, other studies have postulated that prolonged edema results in decreased local cerebral blood flow, which when combined with global hypoperfusion from peripheral shunting of blood can result in ischemic cell death [9,16]. However, the idea that the pathology in these areas of the CNS is related to direct heat injury has not been evaluated. It could be that the pathology in this location is multifactorial.

Similar to previous case reports, our patient showed hyperintense lesions in the cerebellum, frontal and parietal lobes as well as medial thalami [14]. However, to our knowledge there have been few case reports showing diffusion-weighted imaging (DWI) and apparent diffusion coefficient (ADC) mapping in patients with heat stress. Our patient demonstrated increased DWI and decreased ADC signal within the thalami, thus, implicating cytotoxic edema as a potential mechanism of CNS injury early in heat stress.

The MRI changes in our patient did not follow the typical involvement for metabolic disturbances such as hypoglycemia. In hypoglycemia, the temporal lobes, hippocampus, basal ganglia, and substantia nigra appear most susceptible [17]. This topographical preference is well correlated with the T2 changes on brain MRI in hypoglycemic individuals [17]. And although our patient’s restricted diffusion and hyperintense lesions in the cerebellum and thalami are similar to cases of hypoxia-ischemia [18], it is not typical for anoxic-hypoxic injury to have corresponding CNS hemorrhage.

Clinically, the patient’s mental status changes were a consequence of bilateral involvement of the cerebral cortex and thalami affecting the reticular activating system. There are no evidence-based treatments for CNS injury related to heat stress. Given our patient’s improved mental status while on Amantadine, one could question the involvement of dopaminergic systems in heat stress. However, contrary to our experience, animal studies have suggested that survival in heatstroke rats was increased after brain dopamine depletion and inhibition [17].

## Conclusion

Heat stress can cause multiple serious systemic complications ranging from rhabdomyolysis to DIC syndrome to multi-organ failure. However, it is critical to also consider its potential deleterious effects in the CNS. Heat stress should be in the differential diagnosis of patients presenting with encephalopathy especially if the history suggests a preceding episode of hyperthermia and evidence of hyperintense bilateral cerebral, cerebellar, and thalamic lesions. MRI findings in our case are likely multifactorial and attributable to the multiple mechanisms behind heat stress CNS injury. The diffusion hyperintensity, the intraventricular hemorrhage, and the postcontrast enhancement could be secondary to direct heat toxicity, ischemic vasculopathy, blood-CSF breakdown with vasogenic edema, and/or cytotoxic edema. In addition, these changes could be related as a consequence of a heightened inflammatory acute-phase response as shown by elevated serum markers of ESR and CRP.

### Informed consent

Written informed consent was obtained from the patient for publication of this case report and any accompanying images. A copy of the written consent is available for review by the Editor-in-Chief of this journal.

## Competing interests

The authors declare that they have no competing interests.

## Authors’ contributions

WG was involved in drafting and revising the manuscript. SV asssisted with drafting of manuscript. SS and TW were involved in revising the manuscript critically for important intellectual content and have given final approval of the version to be published. All authors read and approved the final manuscript.

## Pre-publication history

The pre-publication history for this paper can be accessed here:

http://www.biomedcentral.com/1471-2377/13/63/prepub
